# The Impact of Rapid Growth After Retardation at the First 1000 Days of Life (FDLs) on the Risk of Noncommunicable Diseases in Indonesian Adolescence

**DOI:** 10.1155/ijpe/4675199

**Published:** 2025-08-07

**Authors:** Ratu Ayu Dewi Sartika, Pika Novriani Lubis, Fadila Wirawan, Edy Purwanto, Ismarulyusda binti Ishak, Dhamas Pratista

**Affiliations:** ^1^Department of Public Health Nutrition, Faculty of Public Health, Universitas Indonesia, Depok, Indonesia; ^2^Department of Epidemiology, Faculty of Public Health, Universitas Indonesia, Depok, Indonesia; ^3^Department of Economics, Faculty of Economics and Business, Airlangga University, Surabaya, Indonesia; ^4^SurveyMETER, Yogyakarta, Indonesia; ^5^Program Sains Bioperubatan, Fakulti Sains Kesihatan, Universiti Kebangsaan Malaysia, Kuala Lumpur, Malaysia

**Keywords:** adolescent, diabetes, growth, hypertension, obesity

## Abstract

**Background:** Rapid growth is commonly found in children after experiencing poor nutritional status. In this context, growth retardation early in life is a major risk factor for developing noncommunicable diseases (NCDs). Therefore, this study is aimed at determining the impact of rapid growth after retardation at the first 1000 days of life (FDLs) on an increased risk of NCD in adolescents.

**Method:** A longitudinal analysis of Indonesia Family Life Survey (IFLS) data was used for 1997, 2000, and 2014, respectively. Meanwhile, the initial data on subjects was taken in 1997 (Age 0–23 months) and observed at 3–5, then 17–19 years. This study used a generalized linear model with a 95% confidence interval for bivariate and multivariate analysis.

**Results:** The results showed a significant association between rapid growth after retardation at the FDLs and an increased risk of hypertension, diabetes mellitus, and obesity in adolescents.

**Conclusion:** The impact of rapid growth after retardation at the FDL increases the long-term risk of NCDs in later life. Early life interventions and policies focused on preventing undernutrition, ensuring a balanced diet, and regularly monitoring growth during the critical period.

## 1. Background

Growth retardation is a common issue in low- and middle-income countries (LMICs) and has been addressed by targeting rapid growth in affected children [1, 2]. However, recent studies reported the potential dangers of the rapid growth pattern often observed in children recovering from undernutrition. The organ development of children subjected to growth retardation may lead to an increased risk of noncommunicable diseases (NCDs) in the subsequent life cycle [3–6]. This pattern is particularly prevalent in LMICs, where the burden of NCDs has risen significantly in recent decades. In Indonesia, 6 out of 10 main causes of death are NCDs. Moreover, the prevalence of NCDs has shifted from initially affecting middle-aged individuals to targeting younger ages because of lifestyle changes in the wider community. Basic health study data showed the prevalence of hypertension (≥ 18 years), obesity (aged 16–18 years), and Type 2 diabetes mellitus (T2DM) increased from 8.7% (2013) to 13% (2018), 1.6% (2013) to 4% (2018), and 10.9% (2018) to 11.7% (2023), respectively. The development of NCDs at a young age tends to be dangerous and can become a complication of more serious diseases [7, 8].

The underlying cause of the phenomenon is related to the “thrifty mechanism” during periods of limited nutrient supply. Poor environments, particularly during the early growth and development phase, induce adaptations for survival. However, adaptation followed by rapid growth, especially within the first 1000 days of life (FDLs), may become maladaptive, predisposing individuals toward obesity, hypertension, and T2DM [9, 10]. This interval age, referred to as “the golden period,” represents a crucial opportunity for intervention.

The interaction between epigenetics, programming early in life, and lifestyle, such as consumption of energy-dense foods and low physical activity, shapes a growth trajectory toward metabolic disease, as the concept of the Developmental Origins of Health and Disease (DOHAD) [10–12]. The results of an Asian cohort study supported this concept, showing that mismatched children experienced the highest accumulation of ectopic fat and increased insulin resistance [5]. A study from Japan concluded that rapid weight gain in the first 3 years of life had two times the risk for dyslipidemia and hypertension in adolescence [13]. We hypothesized that rapid growth is associated with the risk of these diseases. Therefore, this study is aimed at assessing the impact of rapid growth following retardation at FDL on the risk of obesity, T2DM, and hypertension. The analysis of the associations improves the methods for the early prevention and risk reduction of NCDs.

## 2. Methodology

The Strengthening and Reporting of Observational Studies in Epidemiology (STROBE) was followed for reporting this study [14].

### 2.1. Study Design

This study used secondary data from the longitudinal analysis of the Indonesia Family Life Survey (IFLS). IFLS is the only cohort data with the capacity to describe the Indonesian population/society and comprises five waves, with the latest data from 2014 to 2015.

A total of 7224 households taken in 13 provinces from 26 were selected by multistage stratified sampling. The provinces selected as IFLS data samples covered 83% of the population with diversity [15]. The design was retrospective, with data collection in the form of an observational study. The data have been publicly accessible at https://www.rand.org/well-being/social-and-behavioral-policy/data/FLS/IFLS/access.html.

### 2.2. Study Population

Respondents were all children aged 0–23 months (in 1997) who experienced growth retardation and rapid growth in 2000 and were followed until age 17–19 in 2014.

### 2.3. Study Variables

The dependent variables were T2DM, obesity, and hypertension. The study used HbA1c, a glycolytic metabolic compound formed of glucose in the body, hemoglobin, and other proteins, as a marker of T2DM [16]. According to the American Heart Association, the limit for T2DM was ≥ 6.5%, normal (< 5.7%), and pre-DM (5.7%–6.4%) [16]. The HbA1c value was presented in the dried blood spot (DBS) measurement data. A blood drop from the fingertip was collected using Whatman 903 protein saver cards and dried before examination. Body weight and height were measured to calculate BMI based on the formula body weight (kilograms) divided by height (meters) squared. BMI measurement is aimed at observing overweight/obesity status, with a cutoff of ≥ 25 [17]. Weight and height tool measurements were calibrated every day. Systolic blood pressure (SBP) and diastolic blood pressure (DBP) were averaged from three measurements taken in a standard position using an appropriate cuff size on the right/left hand with a sphygmomanometer, Omron HEM-7203. The blood pressure (BP) was considered normal if systolic < 120 mmHg and diastolic < 80 mmHg and was called pre/hypertension if BP ≥ 120 mmHg and ≥ 80 mmHg [18].

The main independent variable was changes in nutritional status (rapid growth after growth retardation in FDL). The measurement of rapid growth was determined by the change in the *z*-score of weight for age (WAZ), height for age (HAZ), and weight for height (WHZ) (between 1997 and 2000, with a cutoff of 0.67 points for children experiencing growth retardation. The WAZ, HAZ, and WHZ were calculated using WHO Anthro and AnthroPlus software for children aged 0–60 months and individuals aged 5–19 years [3]. In the FDL, growth retardation is a condition of prenatal and postnatal malnutrition, measured by birth weight, body weight, and height, as well as grouped according to the criteria of underweight, stunting, and wasting. Meanwhile, different parameters were applied based on previous studies for rapid growth. Desmond and Casale [4] proposed using HAZ. This relative measure is superior to the absolute measure using height-for-age difference (HAD). The latter measure yielded a lower proportion of rapid growth in children than the other measure. Li et al. [19] evaluated rapid growth based on BMI-for-age *z*-score (BAZ), while Ong et al. [5] used rapid weight growth. A systematic review concluded that an increase of 0.67 in the *z*-score indicated a clinical significance in defining rapid growth [20]. Each percentage range in the standard growth chart, such as from the 2nd to 9th percentile, as well as the 9th to 25th, and so forth, has a width of roughly 0.67 SD scores [21].

Covariates were determined based on the previous literature, including the subjects' and parents' characteristics. Subject characteristics comprised sex, education, employment, diet, physical activity, smoking status, working, birth weight, food consumption, and nutritional status of infants and adolescents. For instance, three cohort studies reported that birth weight was associated with growth and BMI, diabetes mellitus, and hypertension [22–24]. The characteristics of the father consisted of education, nutritional status, waist circumference, and history of hypertension and diabetes. Meanwhile, the characteristics of the mother included education, height, waist circumference, and history of hypertension and diabetes. The household characteristics were the type of residence, household size, and amount of household expenditure. Some binary variables included sex (females/males), employment status (not working/working), smoking status (not smoking/smoking), physical activity (active/inactive), height (short/normal), and type of residence (urban/rural). In contrast, the other numeric variables were birth weight (kilograms), education (years of schooling), household size (number of people who occupy a house), household expenditure (percentage), and waist circumference (centimeter) measured with tape to the nearest millimeter.

### 2.4. Statistical Analysis

The dataset was screened for duplication, and descriptive analysis was conducted to present the characteristics of the population as well as the proportion of nutritional status and NCDs. Bivariate and multivariate analyses used generalized linear models (GLMs) presented as coefficients (*β*) with 95% confidence intervals (CIs). Variables having a *p* value < 0.25 were possible confounders and included in the multivariate analysis to determine adjusted *β*. The GLMs analyze a single continuous outcome with multiple independent variables.

In addition, this type of analysis was used when repeated measurements were taken from the same respondent. Multicollinearity and homoscedasticity tests are performed to ensure valid GLM inference, and the missing data was excluded from the analysis. Stata/BE17 (StataCorp LLC) was utilized for all statistical analyses.

### 2.5. Ethics Statement

Our study obtained ethical clearance from Universitas YARSI with Reference Number 017/KEP-UY/EA.10/I/2025. This study used IFLS, a publicly available data that does not require ethical approval. The surveys and procedures received approval from the following ethics committees: the Institutional Review Board (IRB) of RAND Corporation and Universitas Gadjah Mada, under ethical Clearance Number s0064-06-01-CR01. The IRB evaluated the written informed consent obtained from the parents/guardians, which was addressed in the context. Additional information concerning ethical approval can be found at https://www.rand.org/labor/FLS/IFLS.html, and no identifiers have been incorporated in the manuscript.

## 3. Results

### 3.1. Descriptive Analysis

**Figure 1:**
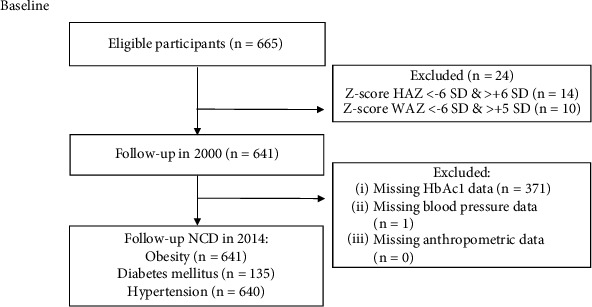
STROBE flow diagram of cohort studies.

A total of 641 babies were recruited in this study (Figure 1 presents more details of the sample selection).  Table 1 shows a descriptive analysis based on the characteristics of children, fathers, mothers, and household conditions. At baseline, the proportion of stunting was 39%, underweight was 23%, wasting was 12%, and low birth weight (LBW) was 22%. At the age of 3–5 years, as many as 78% of babies experienced WAZ rapid growth, 75% experienced HAZ rapid growth, and 71% experienced WHZ rapid growth. After 17 years of follow-up, 20% of children had dysglycemia, 41% had increased SBP, 18% had increased DBP, and 5% were obese.

### 3.2. Bivariate Analysis

The results of the bivariate analysis in Table 2 show that rapid growth in WAZ and WHZ is significantly associated with HbA1c, with crude *β* (95% CI) being 0.881 (0.297; 1.465) and 0.613 (0.077; 1.150), respectively. Of all the covariates, only birth weight and household expenditure were significantly associated with HbA1c. Rapid growth in WAZ and WHZ was significantly associated with BMI, with crude *β* (95% CI) being 1.270 (0.397; 2.144) and 1.100 (0.297; 1.903), respectively. Sex, birth weight, WAZ, and WHZ, as well as SBP and DBP, were significantly related to BMI. Based on parental characteristics, BMI and the waist circumferences of the father and mother are significantly related to BMI in the children.

Rapid growth WAZ was associated with DBP but statistically insignificant in SBP. Sex, education, occupation, smoking status, physical activity, and BMI in adolescents were significantly associated with SBP in adolescence. Regarding the father's characteristics, only SBP was significantly related to all associations. In the mother, education, SBP, and DBP were significantly associated with SBP in adolescence. Meanwhile, sex, HAZ, WHZ, and BMI in adolescents were significantly related to DBP. The SBP and DBP of the mother and father were related to DBP in adolescents. However, there was no significant relationship between the household characteristics and BMI and BP. In this context, rapid WAZ growth was significantly associated with DBP (crude *β* [95% CI] was 2.129 [0.206; 4.051]). HAZ rapid growth was insignificantly related to HbA1c, BMI, and DBP but was significantly correlated to SBP with crude *β* (95% CI) at 3.225 (0.651; 5.798).

### 3.3. Multivariate Analysis

Based on Table 3, an increase of 1 kg in WAZ rapid growth is associated with adjusted *β* HbA1c 0.825 (95% CI 0.227, 1.423) and adjusted *β* BMI 1.403 (95% CI 0.200, 2.607). In addition, an increase of 1 kg in WHZ rapid growth is positively associated with adjusted *β* HbA1c 0.535 (95% CI 0.006, 1.065) and *β* BMI 1.171 (95% CI 0.282, 2.061), while an increase of 1 kg in HAZ rapid growth has a positive association with *β* SBP 3.810 (95% CI 1.590, 8.020) and *β* DBP 1.562 (95% CI 0.161; 3.286), without any significant relationship to HbA1c and BMI.

Birth weight is a significant covariate in the relationship between the rapid growth of WAZ, HAZ, and WHZ on HbA1c. Factors significantly related to the relationship between rapid growth in WAZ and BMI are the short stature of the mother, male infant, HAZ, WHZ, and the father's waist circumference. Stunted mother, father's waist circumference, and WAZ were covariates in the association between rapid growth in WHZ and BMI. In the relationship between HAZ rapid growth and SBP, the covariates with a significant effect are male infant, BMI of adolescent, inactive, smoking, and father's SBP. The male infant, the BMI of the adolescent, and the DBP of the father were confounded in the relationship between HAZ rapid growth and DBP.

## 4. Discussions

This study shows the intricate relationships between rapid growth across key parameters—WAZ, WHZ, and HAZ—and the risks of T2DM, obesity, and hypertension. Rapid growth criteria are based on calculating changes in the growth curve due to the ease of monitoring the system regularly [5].

### 4.1. Rapid Growth as a Predictor of T2DM

The analysis concluded that rapid growth in WAZ and WHZ is a predictor of the incidence of DM. This result is similar to a randomized control trial showing adolescents with increased HbA1c had rapid growth in children [1]. An American cohort study also supported that preadolescent children who experienced rapid weight gain had an increased risk of T2DM [25]. The risk is increased by elevating insulin resistance and/or decreasing insulin sensitivity. This pathogenesis condition is due to decreased muscle mass and changes in the structure and function of pancreatic beta cells triggered by growth retardation [26]. Rapid growth is also marked by elevated C-reactive protein (CRP), a marker of inflammation underlining the occurrence of T2DM [1]. The presence of CRP in obesity signifies chronic inflammation. Moreover, it was suggested that obesity might be an intermediate factor of association between rapid weight gain and T2DM [25, 27]. Fat accumulation due to increased free fatty acids stimulates insulin to store fat. Therefore, this process induces insulin resistance, which decreases the sensitivity, resulting in hyperinsulinemia and changing the structure of the hypothalamus and the function of the sympathoadrenal system. High insulin in the blood also affects glucose output and increases fat oxidation. Therefore, there is a buildup of fatty tissue and a reduction in lean mass [9, 12, 19]. However, this research is aimed at examining the effects of rapid growth on each outcome independently. Another study proved that rapid growth still causes insulin resistance despite adjusting BMI [28].

### 4.2. Rapid Growth as a Predictor of Obesity

The analysis reported that rapid growth in WAZ and WHZ was positively associated with BMI. This is similar to a previous study where babies with rapid weight gain had a higher BMI [24]. Additionally, rapid weight growth in the first 1.5 years of life had a 2.9 times risk of being overweight [13]. The results of a systematic review and meta-analysis stated that rapid weight growth at FDL had the risk of causing obesity [29–31]. An increase in BMI occurs due to rebound adiposity before adolescence [25]. During growth retardation, an energy maintenance process occurs through the thermogenesis process. Therefore, excess energy is formed and stored as fat during rapid growth. Fat is subjected to remodeling as well as changes in gene expression in the tissue. The increase in adiposity is also influenced by leptin resistance and hyperleptinemia, which may be included in biological plausibility [9].

In our study, rapid growth in HAZ was not significantly associated with T2DM and obesity. Previous studies showed that children who were normal (according to HAZ) had an increased risk of T2DM compared to stunted children [22]. Conversely, another study mentioned that stunting was associated with dysglycemia [32]. As known, T2DM was induced by poor eating habits since the first year of life [19, 33]. We suggested that the disparity in outcomes may be associated with unhealthy lifestyles, particularly postchildhood, irrespective of prior stunting. This suggestion was supported by a study in Jamaica, concluding that stunting recovery was not linked with insulin problems. Insulin sensitivity and insulin clearance levels were normal compared to the control group, along with the lack of weight abnormalities in adulthood [34]. A Total Dietary Survey in 2014 indicated that 4.8% of the Indonesian population ingested sugar and 26.5% consumed fat beyond the recommended intake limits [35]. According to the Indonesian Health Survey data in 2023, very few people with T2DM regularly engage in physical activities [8]. Moreover, the questionnaire we used exclusively addressed respondents' activities from the preceding week, thereby neglecting to account for individual habitual patterns. Although the history of physical activity was assessed a week prior, this current engagement may influence the risk of hypertension. A meta-analysis supported this suggestion that newly initiated exercise lowered BP [36].

### 4.3. Rapid Growth as a Predictor of Hypertension

This study reports that only HAZ rapid growth positively affects SBP and DBP, similar to a cohort study in South Africa [37]. This is because changes in BP can occur directly or indirectly through adipose tissue [38]. The analysis supports the presentation that BMI and BP have a significant association. A direct change in BP was associated with changes in axis regulation and heart muscle diameter. Therefore, arterial flow is reduced, and endothelial function is disrupted [39]. This pathogenesis was connected to higher CRP, which may be observed to be higher in rapid growth children with obesity [1].

Although WAZ and WHZ rapid growth were related to BMI in our study, these two parameters were not statistically associated with elevated BP, in contrast with other studies showing that weight gain increased the risk of hypertension [3, 19]. Several studies have found variations in the association between weight gain and hypertension [40–42]. A study conducted in Uganda provided evidence that recovery from wasting led to elevated BP [43], whereas research carried out in Spain reported that rapid weight gain in the first 5 years of life was not associated with hypertension [42]. The biological plausibility was unclear; however, it might be attributed to environmental factors due to poor diet or sedentary behavior.

### 4.4. Strength and Limitation

Concerning the strength of this study, there is no analysis in Indonesia examining the effect of rapid growth after growth retardation at the FDLs on the risk of NCDs in adolescence. Several publications only examined the impact on one type of disease (obesity, T2DM, or hypertension) and used cross-sectional studies. In addition, this longitudinal study provided clinical identification of growth periods based on several parameters. The present result was the first to use multiyear observations or repeated measurement data from IFLS (from 1997, 2000, and 2014 time points), which may aid in constructing a causal relationship. The nondifferential misclassifications of the growth parameters and health outcomes caused negligible selection bias.

However, the study has limitations, such as the lack of data on body fat composition and high missing HbA1c data, affecting the power of statistical analysis and increasing the difficulty of generalizing the entire adolescent population. The evaluation of dietary behavior relied on the self-reported frequency of food intake, without considering portion sizes or caloric content, which resulted in information bias in the nutritional data. In addition, other self-reported questionnaires, including those evaluating smoking and physical activity, were grounded in recent behaviors rather than established habits. Despite the limitations, this study contributes to the literature and serves as a basis for broader analysis with more complete parameters since cohort studies with repeated measurements of the metabolic profile of the population are still rare.

## 5. Conclusion

In conclusion, rapid growth after retardation at FDL was related to the risk of NCDs. A balanced diet and careful regular growth monitoring could be promoted during the critical period to prevent NCDs in later life. Further analysis must also be carried out to determine the effect of rapid growth on NCDs in other periods of life.

## Figures and Tables

**Table 1:** Descriptive characteristics of the population.

**Characteristics**	**1997**	**2000**	**2014**
*Adolescent characteristics*			
Sex (% female)	53.0	—	—
Birth weight (kg)	3.17 (0.51)	—	—
Individuals with LBW (%)	22.0	—	—
HAZ	−1.41 (1.97)	—	—
Individuals with stunting (%)	39.1	—	—
WAZ	−1.01 (1.45)	—	—
Individuals with underweight (%)	22.9	—	—
WHZ	−0.21 (1.62)	—	—
Individuals with wasting (%)	11.8	—	—
BAZ	−0.13 (1.62)	—	—
Individuals with wasting (%)	10.8	—	—
Rapid growth in WAZ (% RG)	—	79.9	—
Rapid growth in HAZ (% RG)	—	75.2	—
Rapid growth in WHZ (% RG)	—	70.8	—
Education (duration in years)	—	—	10.22 (2.16)
Smoking habit (% smoker)	—	—	18.8
Physical active (% inactive)	—	—	69.5
HbA1c (%)	—	—	5.36 (1.02)
Individuals with pre-DM/DM (%)	—	—	20.0
BMI (kg/m^2^)	—	—	20.84 (3.87)
Individuals with obese (%)	—	—	4.5
SBP (mmHg)	—	—	118.14 (12.36)
Individuals with high SBP (%)	—	—	40.9
DBP (mmHg)	—	—	72. 56 (8.75)
Individuals with high DBP (%)	—	—	17.7
*Father's characteristics*			
Education (duration in years)	—	—	7.77 (4.54)
BMI (kg/m^2^)	—	—	23.33 (3.89)
Individuals with obese (%)	—	—	30.8
Waist circumference (cm)	—	—	83.55 (11.32)
Individuals with abdominal obesity (%)	—	—	29.5
HbA1c (%)	—	—	5.90 (1.22)
Individuals with pre-DM/DM (%)	—	—	50.0
SBP (mmHg)	—	—	137.21 (21.70)
Individuals with high SBP (%)	—	—	79.5
DBP (mmHg)	—	—	84.26 (13.42)
Individuals with high DBP (%)	—	—	59.5
*Mother's characteristics*			
Education (duration in years)	—	—	7.16 (4.15)
BMI (kg/m^2^)	—	—	25.61 (4.72)
Individuals with obese (%)	—	—	54.5
Waist circumference (cm)	—	—	85.76 (11.59)
Individuals with abdominal obesity (%)	—	—	67.9
HbA1c (%)	—	—	5.79 (1.35)
Individuals with pre-DM/DM (%)	—	—	38.8
SBP (mmHg)	—	—	132.82 (23.52)
Individuals with high SBP (%)	—	—	66.1
DBP (mmHg)	—	—	82.99 (12.96)
Individuals with high DBP (%)	—	—	52.3
*Household characteristics*			
Annual income (IDR)	—	—	27,982,390 (42,043,592)
Food expenditure (%)	—	—	48.00 (15.20)
Nonfood expenditure (%)	—	—	52.00 (15.22)
Per capita expenditure (IDR)	—	—	1,178,324 (1,025,425)
Household size (number of persons)	—	—	4.43 (2.03)
Living area (% urban)	—	—	64.10

*Note:* Data are presented as mean (SD) for continuous variables and proportion (%) for categorical variables.

Abbreviations: BAZ, BMI-for-age *z*-score; BMI, body mass index; DBP, diastolic blood pressure; DM, diabetes mellitus; HAZ, height-for-age *z*-score; HbA1c, hemoglobin A1c; IDR, Indonesian rupiah; LBW, low birth weight; SBP, systolic blood pressure; WAZ, weight-for-age *z*-score; WHZ, weight-for-height *z*-score.

**Table 2:** The associations of characteristics with the levels of HbA1c, BMI, SBP, and DBP.

**Variables**	**HbA1c levels (%)**		**Body mass index (kg/m** ^**2**^ **)**		**SBP (mmHg)**		**DBP (mmHg)**	
	**β**	**p** ** value (95% CI)**	**β**	**p** ** value (95% CI)**	**β**	**p** ** value (95% CI)**	**β**	**p** ** value (95% CI)**
*Adolescent's characteristics*								
Sex (male vs. female)	0.041	0.815 (−0.303; 0.385)	−0.659	0.031⁣^∗^ (−1.259; −0.059)	12.308	≤ 0.001⁣^∗^ (10.639; 13.976)	1.800	0.009⁣^∗^ (0.446; 3.154)
Birth weight (kg)	0.454	0.020⁣^∗^ (0.072; 0.836)	0.604	0.042⁣^∗^ (0.021; 1.188)	0.555	0.560 (−1.314; 2.425)	−0.567	0.400 (−1.890; 0.756)
HAZ	−0.059	0.188 (−0.146; 0.029)	0.136	0.085 (−0.019; 0.291)	−0.215	0.399 (−0.715; 0.285)	−0.362	0.043⁣^∗^ (−0.713; −0.012
WAZ	−0.075	0.243 (−0.200; 0.051)	0.511	≤ 0.001⁣^∗^ (0.305; 0.716)	0.394	0.246 (−0.272; 1.059)	−0.039	0.871 (−0.512; 0.434)
WHZ	−0.023	0.676 (−0.132; 0.086)	0.260	0.006⁣^∗^ (0.073; 0.447)	0.593	0.054 (−0.011; 1.197)	0.442	0.042⁣^∗^ (0.016; 0.868)
Rapid growth in WAZ (RG vs. non-RG)	0.881	0.003⁣^∗^ (0.297; 1.465)	1.270	0.004⁣^∗^ (0.397; 2.144)	2.642	0.058 (−0.092; 5.377)	2.129	0.030⁣^∗^ (0.206; 4.051)
Rapid growth in HAZ (RG vs. non-RG)	0.027	0.919 (−0.501; 0.556)	0.291	0.486 (−0.529; 1.110)	3.225	0.014⁣^∗^ (0.651; 5.798)	1.544	0.092 (−0.253; 3.340)
Rapid growth in WHZ (RG vs. non-RG)	0.613	0.025⁣^∗^ (0.077; 1.150)	1.100	0.007⁣^∗^ (0.297; 1.903)	1.269	0.331 (−1.295; 3.833)	0.966	0.286 (−0.810; 2.743)
Education (duration in years)	0.065	0.147 (−0.023; 0.153)	0.035	0.626 (−0.105; 0.174)	−0.610	0.007⁣^∗^ (−1.055; −0.164)	−0.225	0.164 (−0.541; 0.092)
Employment status (working vs. unemployed)	−0.111	0.531 (−0.457; 0.236)	−0.338	0.279 (−0.950; 0.275)	2.323	0.020⁣^∗^ (0.374; 4.272)	−0.002	0.998 (−1.387; 1.384)
Smoking status (smoker vs. nonsmoker)	−0.109	0.664 (−0.602; 0.383)	−0.564	0.151 (−1.334; 0.206)	6.085	≤ 0.001⁣^∗^ (3.670; 8.501)	−0.497	0.576 (−2.239; 1.245)
Physical activity (inactive vs. active)	0.114	0.562 (−0.267; 0.496)	−0.135	0.689 (−0.800; 0.529)	−3.500	0.001⁣^∗^ (−5.602; −1.398)	−1.166	0.126 (−2.660; 0.329)
Body mass index (kg/m^2^)	0.028	0.200 (−0.015; 0.071)	—	—	0.968	≤ 0.001⁣^∗^ (0.728; 1.208)	0.656	≤ 0.001⁣^∗^ (0.485; 0.827)
HbA1c level (%)	—	—	0.426	0.205 (−0.236; 1.087)	−3.898	0.734 (−1.869; 2.646)	0.822	0.332 (−0.848; 2.493)
SBP (mmHg)	0.002	0.732 (−0.011; 0.015)	0.092	≤ 0.001⁣^∗^ (0.069; 0.115)	—	—	—	—
DBP (mmHg)	0.009	0.327 (−0.009; 0.026)	0.125	≤ 0.001⁣^∗^ (0.092; 0.157)	—	—	—	—
Number of full meals per day (≥ 3×/day vs. < 3×/day)	−0.039	0.841 (−0.418; 0.340)	−0.480	0.160 (−1.150; 0.190)	2.627	0.106 (0.498; 4.756)	−0.038	0.961 (−1.549; 1.473)
Staple food consumption (day/week)	0.294	0.343 (−0.314; 0.902)	−0.153	0.542 (−0.644; 0.339)	−0.744	0.351 (−2.311; 0.823)	−0.409	0.468 (−1.516; 0.698)
Protein consumption (day/week)	−0.011	0.665 (−0.060; 0.038)	0.031	0.452 (−0.050; 0.113)	−0.014	0.913 (−0.274; 0.245)	0.040	0.671 (−0.144; 0.223)
Vegetable consumption (day/week)	0.053	0.179 (−0.006; 0.112)	−0.008	0.876 (−0.107; 0.091)	−0.182	0.256 (−0.497; 0.133)	−0.172	0.130 (−0.394; 0.051)
Fruit consumption (day/week)	−0.011	0.686 (−0.062; 0.041)	0.018	0.687 (−0.071; 0.107)	−0.243	0.193 (−0.527; 0.041)	0.041	0.692 (−0.161; 0.242)
Sugary food consumption (day/week)	−0.047	0.175 (−0.115; 0.021)	0.022	0.714 (−0.094; 0.137)	−0.566	0.120 (−0.931; −0.201)	−0.234	0.176 (−0.493; 0.025)
Instant noodle consumption (day/week)	−0.032	0.462 (−0.119; 0.054)	≤ 0.001	0.995 (−0.146; 0.147)	0.377	0.114 (−0.091; 0.844)	0.234	0.164 (−0.096; 0.564)
Fast food consumption (day/week)	−0.025	0.739 (−0.173; 0.123)	0.145	0.383 (−0.182; 0.473)	−0.561	0.291 (−1.604; 0.481)	0.352	0.349 (−0.385; 1.088)
Soft drink consumption (day/week)	−0.047	0.454 (−0.172; 0.077)	0.062	0.588 (−0.163; 0.287)	0.597	0.102 (−0.120; 1.314)	0.289	0.264 (−0.218; 0.796)
Fried food consumption (day/week)	−0.021	0.569 (−0.095; 0.052)	0.109	0.171 (−0.009; 0.228)	0.222	0.251 (−0.157; 0.601)	0.206	0.131 (−0.062; 0.474)
*Father's characteristics*								
Education (duration in years)	0.040	0.103 (−0.008; 0.089)	0.027	0.449 (−0.043; 0.096)	−0.201	0.073 (−0.422; 0.019)	−0.006	0.938 (−0.164; 0.152)
BMI (kg/m^2^)	−0.013	0.681 (−0.073; 0.048)	0.331	≤ 0.001⁣^∗^ (0.249; 0.414)	0.277	0.054 (−0.005; 0.559)	0.127	0.209 (−0.071; 0.326)
HbA1c level (%)	0.006	0.962 (−0.230; 0.242)	0.422	0.137 (−0.136; 0.980)	0.791	0.478 (−1.413; 2.995)	0.501	0.533 (−1.089; 2.092)
Waist circumference (cm)	−0.010	0.376 (−0.032; 0.012)	0.086	≤ 0.001⁣^∗^ (0.056; 0.115)	0.061	0.224 (−0.037; 0.159)	0.028	0.428 (−0.041; 0.097)
SBP (mmHg)	0.007	0.208 (−0.004; 0.017)	0.011	0.151 (−0.004; 0.027)	0.062	0.016⁣^∗^ (0.012; 0.112)	0.055	0.002⁣^∗^ (0.020; 0.091)
DBP (mmHg)	−0.002	0.798 (−0.021; 0.016)	0.020	0.118 (−0.005; 0.045)	0.057	0.169 (−0.024; 0.139)	0.087	0.003⁣^∗^ (0.030; 0.144)
*Mother's characteristics*								
Education (duration in years)	0.010	0.633 (−0.033; 0.053)	0.046	0.211 (−0.026; 0.118)	−0.243	0.047⁣^∗^ (−0.482; −0.004)	−0.065	0.451 (−0.233; 0.104)
Height of mother (stunted vs. normal)	0.050	0.777 (−0.293; 0.393)	−0.475	0.121 (−1.077; 0.126)	0.844	0.390 (−1.081; 2.769)	0.381	0.583 (−0.981; 1.744)
BMI (kg/m^2^)	−0.005	0.821 (−0.047; 0.037)	0.188	≤ 0.001⁣^∗^ (0.125; 0.251)	0.047	0.671 (−0.170; 0.264)	−0.041	0.594 (−0.193; 0.111)
HbA1c (%)	0.072	0.439 (−0.110; 0.255)	0.472	0.051 (−0.002; 0.945)	0.944	0.289 (−0.812; 2.700)	1.092	0.092 (−0.180; 2.363)
Waist circumference (cm)	0.003	0.773 (−0.019; 0.026)	0.071	≤ 0.001⁣^∗^ (0.040; 0.102)	0.034	0.505 (−0.066; 0.135)	−0.012	0.730 (−0.080; 0.056)
SBP (mmHg)	−0.006	0.174 (−0.014; 0.003)	0.012	0.065 (−0.001; 0.025)	0.095	≤ 0.001⁣^∗^ (0.052; 0.138)	0.059	≤ 0.001⁣^∗^ (0.028; 0.089)
DBP (mmHg)	−0.010	0.152 (−0.024; 0.004)	0.017	0.145 (−0.006; 0.041)	0.123	0.002⁣^∗^ (0.044; 0.202)	0.128	≤ 0.001⁣^∗^ (0.074; 0.183)
*Household characteristics*								
Living area (urban vs. rural)	0.202	0.257 (−0.148; 0.553)	−0.292	0.360 (−0.919; 0.334)	1.196	0.241 (−0.805; 3.196)	−0.732	0.311 (−2.149; 0.684)
Household size (no. of persons)	−0.008	0.830 (−0.085; 0.068)	−0.016	0.833 (−0.164; 0.132)	−0.251	0.298 (−0.723; 0.222)	−0.314	0.065 (−0.648; 0.019)
Food expenditure (%)	−0.012	0.032⁣^∗^ (−0.023; −0.001)	−0.016	0.111 (−0.036; 0.004)	0.028	0.383 (−0.035; 0.091)	−0.010	0.652 (−0.055; 0.034)
Nonfood expenditure (%)	0.012	0.032⁣^∗^ (0.001; 0.023)	0.016	0.112 (−0.004; 0.036)	−0.028	0.386 (−0.091; 0.035)	0.010	0.667 (−0.035; 0.054)

Abbreviations: *β*, coefficient; BAZ, BMI-for-age *z*-score; BMI, body mass index; CI, confidence interval; DBP, diastolic blood pressure; HAZ, height-for-age *z*-score; HbA1c, hemoglobin A1c; RG, rapid growth; SBP, systolic blood pressure; WAZ, weight-for-age *z*-score; WHZ, weight-for-height *z*-score.

⁣^∗^*p* < 0.05.

**Table 3:** Multivariate analysis of the associations between rapid growth and HbA1c levels, BMI, and blood pressure in adolescents (17–19 years; 2014).

**Rapid growth in WAZ**	**HbA1c levels (%)**		
	***β***	**95% CI**	**p** ** value**

*Intercept*	3.503	1.996; 5.011	≤ 0.001
Rapid growth in WAZ (RG vs. non-RG)	0.825	0.227; 1.423	0.007⁣^∗^
Birth weight (kg)	0.506	0.041; 0.971	0.033⁣^∗^
HAZ (stunted vs. nonstunted)	0.355	−0.082; 0.791	0.111

**Rapid growth in HAZ**	**HbA1c levels (%)**		
	***β***	**95% CI**	**p** ** value**

*Intercept*	3.528	1.912; 5.143	≤ 0.001
Rapid growth in HAZ (non-RG vs RG)	0.013	−0.502; 0.528	0.961
Birth weight (kg)	0.579	0.099; 1.059	0.018⁣^∗^

**Rapid growth in WHZ**	**HbA1c levels (%)**		
	**β**	**95% CI**	**p** ** value**

*Intercept*	3.612	2.068; 5.155	≤ 0.001
Rapid growth in WHZ (RG vs. non-RG)	0.535	0.006; 1.065	0.048⁣^∗^
Birth weight (kg)	0.519	0.036; 1.002	0.035⁣^∗^

**Rapid growth in WAZ**	**Body mass index (kg/m** ^**2**^ **)**		
	**β**	**95% CI**	**p** ** value**

*Intercept*	11.206	7.089; 15.323	≤ 0.001
Rapid growth in WAZ (RG vs. non-RG)	1.403	0.200; 2.607	0.022⁣^∗^
Mother's height (stunted vs. normal)	1.056	0.203; 1.909	0.015⁣^∗^
Gender (male vs. female)	0.852	0.006; 1.697	0.048⁣^∗^
Height for age (*z*-score)	0.304	0.051; 0.558	0.018⁣^∗^
Weight for height (*z*-score)	0.395	0.082; 0.709	0.013⁣^∗^
Father's waist circumference (cm)	0.073	0.038; 0.109	≤ 0.001⁣^∗^
Mother's waist circumference (cm)	0.029	−0.009; 0.067	0.139

**Rapid growth in HAZ**	**Body mass index (kg/m** ^**2**^ **)**		
	**β**	**95% CI**	**p** ** value**

*Intercept*	12.485	9.640; 15.330	≤ 0.001
Rapid growth in HAZ (RG vs. non-RG)	0.173	−0.733; 1.080	0.708
Mother's height (stunted vs. normal)	1.133	0.378; 1.889	0.003⁣^∗^
Father's waist circumference (cm)	0.092	0.059; 0.124	≤ 0.001⁣^∗^

**Rapid growth in WHZ**	**Body mass index (kg/m** ^**2**^ **)**		
	**β**	**95% CI**	**p** ** value**

*Intercept*	13.076	10.104; 16.049	≤ 0.001
Rapid growth in WHZ (RG vs. non-RG)	1.171	0.282; 2.061	0.010⁣^∗^
Mother's height (stunted vs. normal)	1.360	0.574; 2.146	0.001⁣^∗^
Father's waist circumference (cm)	0.083	0.049; 0.117	≤ 0.001⁣^∗^
Weight for age (*z*-score)	0.308	−0.013; 0.628	0.060^+^

**Rapid growth in WAZ**	**SBP (mmHg)**		
	**β**	**95% CI**	**p** ** value**

*Intercept*	111.756	108.765; 114.746	≤ 0.001
Rapid growth in WAZ (RG vs. non-RG)	−0.721	−3.641; 2.200	0.629
Gender (male vs. female)	12.869	10.610; 15.128	≤ 0.001⁣^∗^
BMI of adolescent (kg)	2.839	−3.230; 6.120	0.010⁣^∗^
Smoking (smoker vs. nonsmoker)	4.963	1.570; 8.356	0.004⁣^∗^
Physical activity (inactive vs active)	2.086	−0.375; 4.547	0.097^+^
Father's SBP (mmHg)	0.095	0.047; 0.144	0.024⁣^∗^

**Rapid growth in HAZ**	**SBP (mmHg)**		
	**β**	**95% CI**	**p** ** value**

*Intercept*	105.495	101.157; 109.832	≤ 0.001
Rapid growth in HAZ (RG vs. non-RG)	3.810	1.590; 8.020	0.030⁣^∗^
Gender (male vs. female)	13.376	12.833; 17.919	≤ 0.001⁣^∗^
BMI of adolescent (kg/m^2^)	7.831	3.860; 10.802	0.001⁣^∗^
Physical activity (inactive vs. active)	2.106	−0.355; 4.568	0.094^+^
Smoking (smoker vs. nonsmoker)	4.968	1.574; 8.362	0.031⁣^∗^
Father's SBP (mmHg)	0.039	−0.040; 0.092	0.019⁣^∗^

**Rapid growth in WHZ**	**SBP (mmHg)**		
	**β**	**95% CI**	**p** ** value**

*Intercept*	106.269	101.815; 110.723	≤ 0.001
Rapid growth in WHZ (RG vs. non-RG)	−0.870	−3.461; 1.722	0.411
Gender (male vs. female)	13.609	10.008; 18.209	≤ 0.001⁣^∗^
BMI of adolescent (kg/m^2^)	9.524	7.271; 19.777	≤ 0.001⁣^∗^
Physical activity (inactive vs. active)	2.350	−0.187; 4.887	0.069^+^
Smoking (smoker vs. nonsmoker)	4.280	0.742; 7.817	0.018⁣^∗^
Father's SBP (mmHg)	0.068	0.030; 0.230	0.013⁣^∗^

**Rapid growth in WAZ**	**DBP (mmHg)**		
	**β**	**95% CI**	**p** ** value**

*Intercept*	60.169	55.151; 65.188	≤ 0.001
Rapid growth in WAZ (RG vs. non-RG)	1.759	−3.815; 7.332	0.536
Gender (male vs. female)	1.152	2.505; 11.800	0.003⁣^∗^
BMI of adolescent (kg/m^2^)	3.677	−1.867; 9.880	0.025⁣^∗^
Physical activity (inactive vs. active)	2.989	−1.721; 7.699	0.208
Smoking (smoker vs. nonsmoker)	5.723	−0.804; 12.249	0.104

**Rapid growth in HAZ**	**DBP (mmHg)**		
	**β**	**95% CI**	**p** ** value**

*Intercept*	59.568	55.353; 63.783	≤ 0.001
Rapid growth in HAZ (RG vs. non-RG)	1.562	−0.161; 3.286	0.040⁣^∗^
Gender (male vs. female)	1.814	2.251; 11.376	0.004⁣^∗^
BMI of adolescent (kg/m^2^)	5.916	−1.320; 10.060	0.030⁣^∗^
Physical activity (inactive vs. active)	3.044	−1.692; 7.779	0.208
Smoking (smoker vs. nonsmoker)	5.378	−1.113; 11.869	0.112
Father's DBP (mmHg)	0.059	0.0180; 0.310	0.010⁣^∗^

**Rapid growth in WHZ**	**DBP (mmHg)**		
	**β**	**95% CI**	**p** ** value**

*Intercept*	68.462	49.565; 90.340	≤ 0.001
Rapid growth in WHZ (RG vs. non-RG)	1.857	−2.982; 6.696	0.452
Gender (male vs. female)	1.602	2.2044; 11.159	0.005⁣^∗^
BMI of adolescent (kg/m^2^)	5.909	2.416; 10.343	0.004⁣^∗^
Physical activity (inactive vs. active)	4.279	−0.396; 8.954	0.073^+^
Smoking (smoker vs. nonsmoker)	5.622	−1.247; 12.491	0.109

Abbreviations: *β*, coefficient; BAZ, BMI-for-age *z*-score; BMI, body mass index; CI, confidence interval; DBP, diastolic blood pressure; HAZ, height-for-age *z*-score; HbA1c, hemoglobin A1c; LBW, low birth weight; RG, rapid growth; SBP, systolic blood pressure; WAZ, weight-for-age *z*-score; WHZ, weight-for-height *z*-score.

⁣^∗^*p* < 0.05, ^+^*p* < 0.1.

## Data Availability

The data that support the findings of this study are openly available in IFLS at https://www.rand.org/well-being/social-and-behavioral-policy/data/FLS/IFLS/download.html.
